# The impact of raising a child with a developmental or physical health condition in Ethiopia

**DOI:** 10.1016/j.ridd.2024.104716

**Published:** 2024-05

**Authors:** Anne de Leeuw, Wietske A. Ester, Mersha Kinfe, Fikirte Girma, Rehana Abdurahman, Tigist Zerihun, Atsede Teklehaimanot, Charlotte Hanlon, Hans W. Hoek, Rosa A. Hoekstra

**Affiliations:** a Parnassia Psychiatric Institute, The Hague, the Netherlands; b University Medical Center Groningen, the Netherlands; c Sarr Autism Rotterdam, Youz Child and Adolescent Psychiatry, Rotterdam, the Netherlands; d LUMC-Curium, Child and Adolescent Psychiatry, Oegstgeest, the Netherlands; e Addis Ababa University, College of Health Sciences, School of Medicine, Department of Psychiatry, Addis Ababa, Ethiopia; f Yekatit 12 Hospital Medical College, Addis Ababa, Ethiopia; g St. Paul's Hospital Millennium Medical College, Addis Ababa, Ethiopia; h Neurodiversity Center Ethiopia, Ethiopia; i Tikur Anbessa Specialized Hospital, Addis Ababa, Ethiopia; j King’s College London, Centre for Global Mental Health, Health Service and Population Research Department, Institute of Psychiatry, Psychology and Neuroscience, London, United Kingdom; k Columbia University New York, United States; l King’s College London, Department of Psychology, Institute of Psychiatry, Psychology and Neuroscience, London, United Kingdom

**Keywords:** Africa, Developing countries, Autism, Neurodevelopmental disorders, Quality of life, Family functioning and support

## Abstract

**Objective:**

Raising a child with a developmental disability or physical health condition can have a major impact on the lives of their families, especially in low-income countries. We explored the impact on such families in Ethiopia.

**Study design:**

A total of 241 child-caregiver dyads were recruited from two public hospitals in Addis Ababa, Ethiopia. Of these, 139 children were diagnosed with a developmental disability (e.g. autism, intellectual disability) and 102 children with a physical health condition (e.g. malnutrition, severe HIV infection). The family quality of life was assessed using caregiver reports on the Pediatric Quality of Life Inventory™ (PedsQL-FIM™). The disability weight score, which is a Global Burden of Disease measure to quantify health loss, was estimated for each child.

**Results:**

Families with a child with a developmental disability reported lower quality of life than families caring for a child with a physical health condition (p < .001). Mean disability weight scores in children with a developmental disability were higher than in children with a physical health condition (p < .001), indicating more severe health loss. Disability weight scores were negatively associated with the family quality of life in the whole group (*B=−16.8, SE=7.5, p = .026*), but not in the stratified analyses.

**Conclusions:**

Caring for a child with a developmental disability in Ethiopia is associated with a substantial reduction in the family quality of life. Scaling up support for these children in resource-limited contexts should be prioritized.

## Highlights

● Ethiopian carers of children with developmental disabilities have low quality of life.
● Their quality of life is lower than carers of children with physical health problems.
● Child health loss is associated with a lower family quality of life.
● Scaling-up of support for these families deserves prioritization.

## What this paper adds

### Article Summary

This comparative study explores the family impact of raising children with developmental disabilities or physical health conditions in Ethiopia, making use of disability weight scores.

## What’s known on this subject

Caring for children with developmental disabilities or physical health conditions is associated with a reduced family quality of life. Most previous studies have been done in high-income countries, while insights from very low-income contexts are lacking.

## What this study adds

Ethiopian caregivers raising children with developmental disabilities reported a lower family quality of life than those whose children had physical health conditions. Child health loss was associated with a lower family quality of life. Scaling-up family support needs prioritization.

## 1 Introduction

Caring for a child with a developmental disability or physical health condition can have a profound impact on the lives of families and caregivers. These caregivers report higher perceived stress levels (Ooi et al., 2016, Francisco Mora et al., 2020), more sleep deprivation (Ooi et al., 2016, Isa et al., 2016) and a poorer quality of life (Francisco Mora et al., 2020, Isa et al., 2016, Macedo et al., 2015, Vasilopoulou and Nisbet, 2016) compared to caregivers in the general population. In low-income countries, the impact of caring for a child with a disability is especially high (Thrush & Hyder, 2014) with formal support, including access to education or targeted interventions often limited in these contexts (Zeleke et al., 2021, Tekola et al., 2016, Tilahun et al., 2019, Gebeyehu et al., 2019, Ademosu et al., 2021, McKenzie and McConkey, 2015).

Most research on developmental disabilities has been conducted in high-income, mainly Western countries (Durkin et al., 2015, de Vries, 2016, Roy et al., 2021), while only around 5% of the young children with a developmental disability live in these countries (Global Research on Developmental Disabilities Collaborators, 2018). Studies from low-income countries have reported barriers to care, such as financial constraints, war or food insecurity (World Health Organization, 2008) and limited availability and geographical accessibility of adequate health services (de Leeuw et al., 2020). Similar challenges were also identified amongst caregivers of children with physical health conditions. For instance, socioeconomic circumstances and child food insecurity were associated with increased stress levels, psychological morbidity, and even suicidal ideation amongst caregivers of children with HIV in South Africa and Malawi (Skeen et al., 2014). In addition, families caring for children with disabilities in low-income countries may experience high levels of stigma (Ademosu et al., 2021, Tornu et al., 2023, Jansen-van Vuuren et al., 2022), especially those who have children with developmental disabilities (Ademosu et al., 2021, Jansen-van Vuuren et al., 2022). Although caregiver support is often given by extended family and the local community (Thrush & Hyder, 2014), high levels of stigma can result in the family being isolated from this social support (Ademosu et al., 2021, Jansen-van Vuuren et al., 2022).

These barriers to support have also been shown in Ethiopia, where the availability of health care services and psychiatrists is limited (Tekola et al., 2016, Tilahun et al., 2019, Gebeyehu et al., 2019), caregivers feel disempowered through poverty (Szlamka et al., 2023), access to appropriate education is poor (Zeleke et al., 2021), and high stigma has been reported (Tekola et al., 2020).

We examined the family impact of caring for a child with a developmental disability (including autism spectrum disorder, attention deficit hyperactivity disorder, developmental language disorder, and intellectual disability) compared to a child with a physical health condition in Ethiopia. The broad range of physical health conditions in this sample represents the variety of diagnoses seen in a pediatric clinic of a governmental Ethiopian hospital. The impact of caring for a child with a physical health or developmental condition was explored by assessing the caregiver-reported family quality of life, and international standardized Global Burden of Disease Disability Weight Scores, which quantify the severity of health loss (Global Burden of Disease Collaborative Network, 2019). Our research questions were: (1) What is the quality of life in families of children with developmental disabilities compared to families of children with physical health conditions? (2) What is the estimated health loss in a representative help-seeking sample of children with developmental disabilities compared to children with physical health conditions? (3) What is the association between family quality of life and health loss?

## 2 Methods

### 2.1 Study design

This cross-sectional study was conducted in the capital of Ethiopia, Addis Ababa. Participants were recruited in two government hospitals: the Yekatit 12 Hospital Medical College and the St. Paul’s Hospital Millennium Medical College. Both hospitals have a general pediatrics clinic and a child mental health clinic. Participants were consecutively recruited from families seeking help at either clinic. Data were collected over 10 months (August 2018 to May 2019). Informed consent was obtained from all participants. Participants were financially compensated for their time and any additional travel costs. Nurses who worked independently of the patients’ clinicians were trained as data collectors. The nurses conducted face-to-face interviews for all participants because of variable literacy levels. Additional information was extracted from the medical records, including information on the child’s clinical diagnosis, date of diagnosis, date of hospital visit, and any medication prescribed.

### 2.2 Participants

**Fig. 1:**
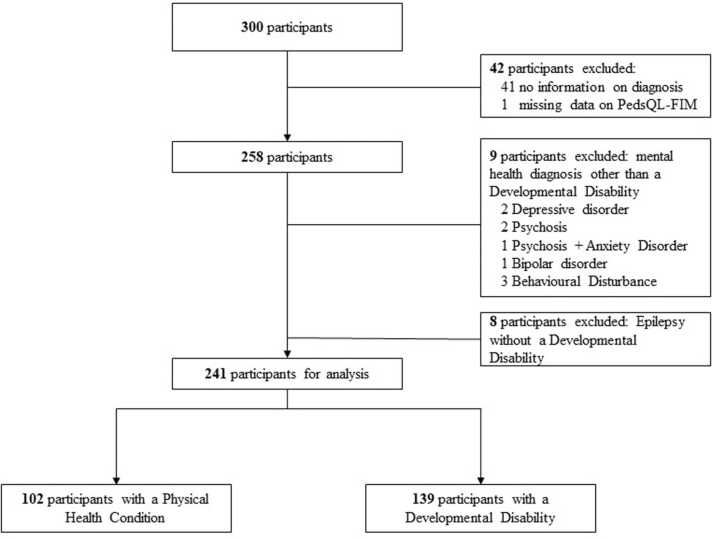
Flowchart of the study population.

The children were divided into two groups, based on their formal clinical diagnosis: those diagnosed with a physical health condition, and those with one or more of the following developmental disabilities: autism spectrum disorder, attention deficit hyperactivity disorder, developmental language disorder or intellectual disability. In total, 300 children and their caregivers were enrolled in the study (Fig. 1). Of these, 59 children were excluded because the family quality of life questionnaire was incomplete (n = 1), the formal clinical diagnosis was missing (n = 41), they had a mental health diagnosis without a developmental disability (n = 9), or an epilepsy diagnosis without a developmental disability (to avoid mischaracterizing children in whom a developmental disability was not (yet) diagnosed n = 8). The final sample comprised 241 mother-child dyads, including 139 children with a developmental disability and 102 children with a physical health condition.

#### 2.2.1 Physical health condition group

The primary reason for a child with a physical health condition to visit the outpatient clinic was due to concerns ranging from a mild anemia to severe pneumonia or an HIV infection (Table S1). As the data was collected among consecutively recruited help-seeking families, the range of conditions is reflective of the wide range of conditions seen in these two state-owned pediatric clinics. To explore whether any group differences observed between the developmental disabilities and physical health conditions group may be explained by a subgroup of physical health patients with short-term conditions that have a lesser impact on family functioning, we performed a subgroup analysis only including children with chronic health condition diagnoses (n = 57). The chronical health conditions list (included in Table S1) was developed by (Ethiopian) medical specialists (coauthors AT, WE, HWH, AdL ) who were masked for the disability weight scores associated with specific diagnoses when developing the list.

#### 2.2.2 Developmental disability group

Clinical diagnoses of developmental disabilities were provided in child mental health clinics by general psychiatrists without specialist expertise in child psychiatry, since this training was not available in Ethiopia until 2022. The diagnoses were made according to the Diagnostic and Statistical Manual of Mental Disorders (American Psychiatric Association, 2013) and were based on neurodevelopmental assessments and the psychiatrists’ clinical judgement. The diagnostic assessments in the child mental health clinics focused on mental and developmental health; the physical health of participants in this group was not systematically and comprehensively assessed. Therefore, we cannot exclude the possibility that some children in this sample also had co-occurring physical health needs.

### 2.3 Variables

#### 2.3.1 Quality of life

To assess family quality of life, we used the Pediatric Quality of Life Inventory, Family Impact Module (PedsQL-FIM), acute version (Varni et al., 2004). This questionnaire was developed in English and then translated and validated for use in various countries across the world (Varni et al., 2004, Rahman et al., 2011, Chen et al., 2011, Knez et al., 2015, Scarpelli et al., 2008). Recently, the questionnaire was culturally adapted and validated for use in Ethiopia, where the hypothesized 8-factor structure showed an acceptable model fit and the measure demonstrated high internal consistency, good test-retest reliability, and high known group validity (Borissov et al., 2021).

The PedsQL-FIM comprises 36 items and has eight subscales with questions reflecting on the past seven days: physical functioning (6 items), emotional functioning (5 items), social functioning (4 items), cognitive functioning (5 items), communication (3 items), worry (5 items), daily activities (3 items) and family relationships (5 items). The total score was computed by averaging all 36 items. A parental Health-Related Quality of Life (HRQL) summary score was computed from the items in the physical, emotional, social and cognitive functioning scales. A summary score of family functioning was computed from the average of the items in the daily activities and family relationships scales. All items were assessed on a 5-point Likert scale ranging from 0 *Never*, to 4 *Almost always*. The item scores are subsequently reverse-scored and rescaled to 0, 25, 50, 75 and 100, with an average score of 100 indicating perfect quality of life. One participant had more than 25% missing items and was excluded from our analyses; the remaining participants completed all the individual items. In the current sample, the PedsQL has excellent internal consistency (PedsQL total score in the Developmental Disabilities group: α = .94, in the physical health conditions group: α = .96).

#### 2.3.2 Health loss

The severity of health loss associated with different developmental- and health conditions was estimated using Global Burden of Disease disability weight scores (Global Burden of Disease Collaborative Network, 2019). This measure quantifies loss-of-function for all non-fatal consequences of disease or injury. The disability weight score lies between 0 (no disability) and 1 (death). There is evidence of highly consistent results across samples from different cultural environments (Global Burden of Disease Collaborative Network, 2019, Salomon et al., 2012). Clinical diagnostic information and medication prescription (taken from medical records) were used to define the diagnosis and subsequently assign a standardised disability weight score to each child participant. In addition, we evaluated information on the child’s age, date of diagnosis, the date of hospital visit, and responses to the Communication Profile-A (Ceccarelli et al., 2021) (assessing a child’s communication ability) and the Autism Treatment Evaluation Checklist (ATEC) (Borissov et al., 2021) (assessing developmental and behavioral functioning) when assigning individual disability weight scores. Barriers to healthcare access mean that most patients in the two governmental clinics present with severe developmental- or health problems. Therefore, the disability weights in this study were consistently scored as ‘severe’, unless specific information was available to suggest a lesser severity. If a participant had more than one diagnosis for which there was no special combined disability weight score, the diagnosis with the highest score was used. As almost all the children with a formal autism diagnosis presenting in the clinic also have an intellectual disability, we used the combined disability weight scores of autism and intellectual disability in our analysis (Global Research on Developmental Disabilities Collaborators, 2018).

The disability weights were assigned to each individual by two clinicians WE and AdL. Any questions arising from their medical records were discussed case by case with Ethiopian clinical specialists FG, TZ, RA after which the most suitable disability weight score was agreed upon).

#### 2.3.3 Demographic Data

Caregiver-reported demographic data of both the caregiver and child was collected (Table 1), including caregiver’s age, sex, marital status, level of education, area of residence, occupation and religion, as well as the child’s age and sex.

**Table 1:** Characteristics of participating caregivers and children by type of diagnosis.

		Developmental Disability	Physical Health Condition
		*n = 139*	*n = 102*
**Caregiver characteristics**			
Age at intake in years, mean (SD)		34.95 (7.66)	33.62 (6.24)
	Missing	0.0	0.0
Gender (% female)*		89.9	71.6
	Missing	0.0	0.0
Relationship to the child (%)*			
	Mother	83.5	69.6
	Father	9.4	26.5
	Extended family	3.6	3.9
	Other caregiver	2.2	0.0
	Missing	1.4	0.0
Education level (%)			
	No formal education	14.4	16.7
	Primary school	30.2	36.3
	Secondary school	33.8	31.4
	Secondary school diploma	7.9	6.9
	College	12.2	7.8
	Missing	1.4	1.0
Occupation (%)			
	Farmer	3.6	4.9
	Housewife	51.8	35.3
	Merchant	11.5	11.8
	Civil servant	4.3	6.9
	Daily laborer	7.9	16.7
	Student	0.0	1.0
	Other	2.9	6.9
	Missing	18.0	16.7
Marital status (%)			
	Married	79.9	86.3
	Single	3.6	2.9
	Divorced	12.9	7.8
	Widowed	2.9	2.9
	Missing	0.7	0.0
Area of residence (% urban)		84.9	78.4
	Missing	2.2	1.0
Religion (%)*			
	Orthodox Christian	56.8	68.6
	Protestant	7.9	12.7
	Catholic	0.7	1.0
	Muslim	33.8	15.7
	Other (Waaqeffanna)	0.0	2.0
	Missing	0.7	0.0
**Child characteristics**			
Age at intake in months, mean (SD)* **		64.10 (22.03)	49.11 (23.62)
	Missing	0.0	0.0
Gender (% girls)* **		25.2	47.1
	Missing	0.0	1.0

Note: Continuous variables are presented as means and standard deviations (SD). Categorical variables and missing data are presented as percentages. P-values are derived from t-tests for continuous variables and chi-square tests for binary/categorical variables.

* Statistically significant (p < 0.05), * ** Statistically significant (p < 0.001)

### 2.4 Statistical analyses

Statistical analyses were performed using the Statistical Package of Social Sciences (SPSS), version 27.0 for Windows (IBM SPSS Statistics for Windows.). Descriptive statistics were generated for all caregivers and their children by type of diagnosis. Missing values in covariates were handled using Multivariate Imputation by Chained Equations (MICE) (Van Buuren, 2018), with 20 imputed datasets. As a sensitivity analysis, the analyses were also conducted on complete cases. Group differences in caregiver-reported family functioning (PedsQL-FIM) between the developmental disability and physical health condition groups were studied using analysis of covariance (ANCOVA), including as covariates: caregiver education, age and gender, and the child’s age and gender. The interaction between all covariates and the treatment variables (diagnosis) in the association of diagnosis with PedsQL was tested using the ‘Test for Treatment by Covariate Interaction’ function in SPSS.

Group differences in disability weight scores between both groups were studied using ANOVA. Disability weight scores are standardised measures and thus we did not account for covariates. A regression analysis was performed to study the association between disability weight scores and caregivers’ reported family functioning.

## 3 Results

### 3.1 Population description

Demographic characteristics of the caregivers and their children are shown separately for the developmental disability (n = 139) and physical health condition (n = 102) groups (Table 1). Caregivers had similar ages in the two groups (mean age 34 and 35 years, respectively). The majority of reporting caregivers were women (developmental disability group 90% vs physical health condition group 72%; p < .05). Children in the developmental disability group were significantly older on average than children with physical health conditions (mean age 64 vs 49 months; p < .001). Children in the developmental disability group were more often male (developmental disability 75% vs physical health condition 53%, p < .001). Correlations between Maternal and Child Characteristics, and PedsQL-FIM sum scores are shown in Supplementary Table 2. Caregiver quality of life was significantly associated with caregiver level of education in the total group (r = .26, p = .001) as well as in both subgroups (r = .25, p = .003 in the developmental disabilities group; r = .33, p = .001 in the physical health group). Caregiver quality of life was significantly negatively associated with caregiver age in the total group (r = −.17, p = .008) and in the developmental disability group alone (r = −.20, p = .021), but not in the physical health group (p = .405). Similarly, caregiver quality of life was significantly negatively associated with child age in the total group (r = −.15, p = .024) and in the developmental disability group alone (r = −.21, p = .013), but not in the physical health group (p = .310).

### 3.2 Family quality of life and disability weight scores

Mean scores on the PedsQL-FIM and mean disability weight scores are reported in Table 2. Families of children with a developmental disability had lower mean scores on the PedsQL-FIM, indicating a lower quality of life compared to families of children with a physical health condition (mean developmental disability 53.8 vs physical health condition 64.3; p < .001). Caregiver education was a significant covariate in the model (education caregiver: B =6.15, SE =0.97; p < 0.001); the other covariates were not significant (age caregiver: B =−0.31, SE =0.16, p > .05; gender caregiver: B =−0.13, SE =2.98, p > .05; age child: B =0.01, SE =0.05, p > .05; gender child: B =−1.24, SE =2.36, p > .05. Diagnostic group alone (not accounting for covariates) explained around 6% of variation in PedsQL-FIM total scores (R Square =.066, Adjusted R Square =.063, F(6,234) = 18.63, t(235) = −4.33; No significant interactions were found between the dependent variable (diagnostic group) and the covariates in the association between diagnosis and PedsQL-FIM. Similar overall group differences were found when separately focusing on the PedsQL FIM HRQL summary scale (mean score 54.6 vs 65.0, respectively, p < .001) and the family functioning summary scale (mean score 60.0 vs 69.1, respectively, p = .007).

**Table 2:** Quality of Family Life sum scores (PedsQL-FIM) and Disability Weight Scores by type of child diagnosis.

		Developmental Disabilities (n = 139)					Physical Health Conditions (n = 102)ᴬ						Physical Health Conditions, chronic only (n = 57)ᴮ					
				*95% CI*					*95% CI*						*95% CI*			
		*Mean*	*SE*	*LL*	*UL*	*α*	*Mean*	*SE*	*LL*	*UL*	*α*	*p*	*Mean*	*SE*	*LL*	*UL*	*α*	*p*
**PedsQL-FIM**																		
Total Score		53.81	1.48	50.91	56.71	.94	64.29	1.76	60.84	67.73	.96	< .001	63.18	2.47	50.73	56.70	0.96	.001
	HRQL	54.56	1.57	51.48	57.63	.91	64.97	1.86	61.31	68.62	.95	< .001	64.60	2.61	60.27	70.57	0.95	< .001
	Family Functioning	60.03	1.97	56.16	63.89	.90	69.09	2.34	64.50	73.67	.91	.007	69.36	3.38	63.09	76.43	0.91	.016
**Disability Weight**																		
Total Score		0.28	0.01	0.26	0.30	-	0.18	0.02	0.14	0.20	-	< .001	0.20	0.02	0.17	0.24	-	.002

Note. p-values are based on analysis of covariance (ANCOVA) for the PedsQL-FIM analyses. Results presented are adjusted for age, gender and education of the caregiver, and age and gender of the child. Education of the caregiver was the only significant covariate (p < 0.001). p-values were derived from analysis of variance (ANOVA) for the Disability Weight Score analyses. p-values concern the group comparison to the ''Developmental Disabilities'' group. HRQL=Health Related Quality of Life, Mean=adjusted mean, SE=standard error, 95% CI= 95% confidence interval; LL=lower limit; UL=upper limit, α = Cronbach’s alpha.

ᴬSample size is n = 102 for the PedSQL, and n = 98 for the Disability Weight analysis (4 cases had missing Disability Weight Scores). ᴮSamples size is n = 57 for the PedSQL analysis and n = 54 for the Disability Weight analysis (3 cases had missing Disability Weight Scores).

Higher mean disability weight scores, indicating more severe health loss, were observed in the developmental disability group compared to the physical health condition group (mean 0.28 vs 0.18, respectively; p < .001). The sub analysis comparing the developmental disability group with children with chronic physical health conditions showed similar results (Table 2). Diagnostic group explained around 12% of the variance in disability weight scores (R Square = 0.118, Adjusted R Square =0.114, F(1,235) = 31.49, t (236) = 5.61.

### 3.3 Association of Disability Weight Scores with Family Quality of Life Scores

Results of our regression analyses of disability weight scores and PedsQL-FIM scores are reported in Table 3. Considering both groups together, disability weight scores were negatively associated with PedsQL-FIM total scores (B = −16.9, SE =7.6, p = .026, CI = [−31.8, −2.0]), indicating that caregivers of children with a diagnosis associated with more severe health loss reported a lower quality of life. Within the group of children with a developmental disability, a higher degree of health loss was associated with lower HRQL sub-scores (B = −21.0, SE = 10.6, p = .048, CI = [−41.8, −0.2]). The associations were not significant in the family functioning sub-score, nor in the total score of the developmental disability group. Neither did they reach significance in the physical health condition group in isolation. Findings of all analyses were similar when only complete cases were considered. Similar results were also found in the sub analysis in the group of children with a chronic physical health condition.

**Table 3:** Association of Disability Weight Scores with Quality of Family Life scores (PedsQL-FIM).

		Total group (n = 237)					Developmental Disability (n = 139)					Physical Health Conditions (n = 98)ᴬ					Physical Health Conditions, chronic only (n = 54)ᴮ				
					95% CI					95% CI					95% CI					95% CI	
		*B*	*SE*	*p*	*LL*	*UL*	*B*	*SE*	*p*	*LL*	*UL*	*B*	*SE*	*p*	*LL*	*UL*	*B*	*SE*	*p*	*LL*	*UL*
**PedsQL -FIM**																					
Total Score		-16.88	7.59	.026	-31.75	-2.02	-17.47	10.23	.088	-37.53	2.58	10.15	12.80	.428	-14.98	35.27	25.51	15.5	.144	-87.49	59.76
	HRQL	-14.74	8.08	.068	-30.58	1.11	-21.0	10.62	.048	-41.82	-0.17	21.82	14.04	.120	-5.72	49.36	37.38	19.31	.052	-0.47	75.23
	Family Functioning	-15.31	10.08	.129	-35.06	4.44	-18.10	15.32	.238	-48.13	11.94	12.32	13.56	.364	-14.30	38.95	20.69	19.4	.287	-17.34	58.72

Note. p-values are derived from multiple regression analyses. Results are given after adjusting for age, gender and education of the caregiver, and age and gender of the child. HRQL=Health Related Quality of Life, B=unstandardized effect, SE=standard error, 95% CI= 95% confidence interval; LL=lower limit; UL=upper limit. ᴬ4 cases had missing Disability Weight Scores, ᴮ3 cases had missing Disability Weight Scores.

Disability Weight Scores and covariates explained 21% of variance in PedsQL-FIM total scores in the total group (R square =0.21, F (6,230) = 10.25, t (231) = −2.23. Covariates: age caregiver: B = −0.41, SE = 0.17, gender caregiver: B = 3.83, SE = 2.91, education caregiver: B = 5.73, SE = 0.99, age child: B = −0.04, SE = 0.05, gender child: B = −3.19, SE = 2.37).

## 4 Discussion

In this study we show that caring for a child with a developmental disability in Ethiopia was associated with a substantially poorer reported family quality of life compared to caring for a child with a physical health condition. In our sample groups, the estimated disability weight scores were also higher for children with a developmental disability compared to children with a physical health condition, indicating more severe health loss. Disability weight scores were negatively associated with family quality of life in the whole sample, but the association was no longer significant when we considered the two groups of children separately. These results are similar when analyzing only physical health conditions with a more enduring, chronic character.

Our quantitative study is the first to show a reduction of family quality of life when a child has a developmental disability compared to a physical health condition in a low-income country. The profound impact on the lives of caregivers and families when raising a child with a developmental disability has, however, been reported in studies from high-, as well as low- and middle-income countries (Vasilopoulou and Nisbet, 2016, Ademosu et al., 2021, Jansen-van Vuuren et al., 2022, Tekola et al., 2020, Gona et al., 2016, Masulani-Mwale et al., 2016).

In this sample from Ethiopia, caregiver educational level was positively associated with family quality of life. This result is in line with previous studies on families of children with developmental disabilities (Chou et al., 2007) or chronic diseases (Toledano-Toledano, 3 et al., 2020). Educational level is likely a proxy for socio-economic status and access to care and this may explain the moderate association between education and quality of life. Many families in Ethiopia still have limited access to education, as illustrated by the high percentage of caregivers in our sample who had received no formal education or only primary education.

Quality of life was also modestly negatively associated with child and caregiver age in the developmental disabilities group. One explanation for this finding is that the enduring burden of caring for a child with a developmental disability has an increasing impact on the family’s life, for example because of enduring social isolation and poverty (Szlamka et al., 2023, Tekola et al., 2020).

In our consecutively recruited sample of participants from two government hospitals, we estimated more severe health losses in the children with a developmental disability than in those with a physical health condition. To place the mean disability weight scores in a broader perspective: the mean disability score of the group with a developmental disability (0.28) is comparable to the somatic diagnoses of ‘cancer, diagnosis and primary therapy (0.29)’ and ‘diabetic neuropathy with untreated amputation (0.28)’. The mean disability weight score of the physical health group (0.18) is comparable to the diagnoses of ‘kwashiorkor and severe wasting (0.17)’ and ‘vertigo with moderate hearing loss and ringing (0.18)’ (Global Burden of Disease Collaborative Network, 2019).

The universality of disability weight scores across different cultural settings or contexts has been discussed extensively (Salomon et al., 2012). What can be rated as less severe in one cultural setting or context might be rated as more burdensome in a different setting. However, in the most recent Global Burden of Disease Disability Weight studies, participating countries were selected for diversity in language, culture and socioeconomic status. As stated in the Global Burden of Disease Study 2010: ‘By contrast with the popular hypothesis that disability assessments vary widely across samples with different cultural environments, we have reported strong evidence of highly consistent results’ (Salomon et al., 2012).

Our study shows that more severe health loss in the child was related to a lower family quality of life in the whole group. In children with a developmental disability, more severe health loss was related to a lower Health- Related Quality of Life reported by caregivers. Given the high levels of stigma when raising a child with a developmental disability in Ethiopia and other sub-Saharan countries (Ademosu et al., 2021, Tekola et al., 2020), the universal disability weight scores used in our study might underestimate the true degree of health loss experienced in Ethiopia related to developmental disabilities.

### 4.1 Strengths and limitations of this study

A strength of our study is that data were collected from a representative sample of families seeking medical help in Addis Ababa. Most participants had limited education, reflecting the low-income context in Ethiopia, and providing insights from a vulnerable population strongly underrepresented in the international developmental health literature (Durkin et al., 2015). In addition, we used a quality of life questionnaire that was adapted and validated in the Ethiopian context (Borissov et al., 2021). Our participating sample covered physical health conditions as well as developmental disabilities, thereby yielding results from two groups that are often studied separately.

One limitation of our study was the reliance on medical records to define the diagnosis and its severity. Also, we did not have exhaustive clinical information about physical health conditions that may have affected the children in the developmental disabilities group. However, since this group was found to have a greater average disability weight score compared to the physical health condition group, any co-occurring physical health conditions in the developmental disability group that this study failed to take into account are unlikely to have affected the overall findings of this study. Lastly, we conducted our study in Addis Ababa, in a largely urban population, and this may not be representative for the Ethiopian population as a whole.

## 5 Conclusion

Our results show that families raising children with a developmental disability in a low-income, sub-Saharan country report a reduced quality of life compared to those raising children with a physical health condition. Caregivers of children with more severe health loss reported a lower family quality of life. Quality of life was especially compromised in families with low levels of education, highlighting their vulnerable position in society. Almost 53 million young children live with a developmental disability worldwide, with roughly 95% living in low- and middle-income countries (Global Research on Developmental Disabilities Collaborators, 2018). It is therefore critical that we better address the needs of these children and their caregivers. Both healthcare and educational support must be scaled-up to create an enabling and healthy environment for the whole family.

## Ethical considerations

The study was approved by the College of Health Sciences Institutional Review Board at Addis Ababa University (062/16/Psy) and by the Psychiatry, Nursing and Midwifery Research Ethics Subcommittee, King’s College London (HR-16/17 – 3489).

## Funding/support

This work was supported by funding from four United Kingdom organizations: the Medical Research Council (MRC), the Department for International Development (10.13039/501100000278DFID), the Welcome Trust, and the National Institute for Health Research (10.13039/100006662NIHR) (#MR/P020844/1). FG, RA, TZ, CH and RAH are supported by funding from the National Institute for Health and Care Research (NIHR200842) and CH also through NIHR134325 using UK aid from the UK government. The views expressed in this publication are those of the authors and not necessarily those of the NIHR or the UK Department of Health and Social Care. CH is also funded by the Welcome Trust through grants 222154/Z20/Z (SCOPE) and 223615/Z/21/Z (PROMISE). For the purpose of open access, the author(s) has applied a Creative Commons Attribution (CC BY) licence to any Author Accepted Manuscript version arising.

## Data Availability

Data will be made available on request.
