# Supplementary material for: The impact of raising a child with a developmental or physical health condition in Ethiopia
Source: Res Dev Disabil. 2024 May;148:104716. doi: 10.1016/j.ridd.2024.104716 (PMC11413522; doi:10.1016/j.ridd.2024.104716)
Supplement: Supplementary file 1 — Supplementary material [file mmc1.docx]

| **TABLE S1 Overview of Physical Health Conditions in our Sample of 102 Children** | | | |
| --- | --- | --- | --- |
| **Diagnosis** | **Number of individuals** | | **Percentage of our Sample** |
| Pneumonia | | 23 | 19.3 |
| Severe Acute Malnutrition* | | 9 | 7.6 |
| Congestive Heart Disease* | | 7 | 5.9 |
| Hepatitis | | 6 | 5.0 |
| Upper Respiratory Tract Infection | | 6 | 5.0 |
| Acute Gastroenteritis | | 6 | 5.0 |
| Anemia* | | 5 | 4.2 |
| Human Immunodeficiency Virus* | | 5 | 4.2 |
| Asthma | | 3 | 2.5 |
| Cyst* | | 3 | 2.5 |
| Meningitis | | 3 | 2.5 |
| Undescended Testis* | | 3 | 2.5 |
| Rickets* | | 3 | 2.5 |
| Hyperactive Airway Disease* | | 3 | 2.5 |
| Parasite* | | 2 | 1.7 |
| Nephritic Syndrome* | | 2 | 1.7 |
| Nephrotic Syndrome* | | 2 | 1.7 |
| Tuberculosis* | | 2 | 1.7 |
| Bronchitis* | | 2 | 1.7 |
| Acute Tonsillopharyngitis | | 1 | 0.8 |
| Acute Febril Illness | | 1 | 0.8 |
| Acute Glomerulonephritis | | 1 | 0.8 |
| Allergic Rhinitis | | 1 | 0.8 |
| Bilateral Leg Deformity* | | 1 | 0.8 |
| Epistaxis | | 1 | 0.8 |
| Hypercalcemia, Hypertension* | | 1 | 0.8 |
| Hyperkalemia | | 1 | 0.8 |
| Hypoalbuminemia | | 1 | 0.8 |
| Juvenile Rheumatoid Arthritis | | 1 | 0.8 |
| Femoral Shaft Fractures | | 1 | 0.8 |
| Mesenteric Lymphadenitis | | 1 | 0.8 |
| Underweight | | 1 | 0.8 |
| Basal Skull Fracture | | 1 | 0.8 |
| Myalgia* | | 1 | 0.8 |
| Obstructive Sleep Apnea* | | 1 | 0.8 |
| Pediatric Stroke | | 1 | 0.8 |
| Pertussis | | 1 | 0.8 |
| Chest Hemangioma* | | 1 | 0.8 |
| Hydrocele* | | 1 | 0.8 |
| Hemiparesis* | | 1 | 0.8 |
| Syncope | | 1 | 0.8 |
| Diabetes Mellitus* | | 1 | 0.8 |
| Circumcision* | | 1 | 0.8 |
| **Total** | | **119** | **100.0** |
| Note. Some children had more than one diagnosis:15 participants had two diagnoses and two participants had three diagnoses. In four cases, no adequate Disability Weight Score could be determined. *Classified as a chronic diagnosis by (Ethiopian) Medical Specialists. | | | |
