# Supplementary material for: The impact of raising a child with a developmental or physical health condition in Ethiopia
Source: Res Dev Disabil. 2024 May;148:104716. doi: 10.1016/j.ridd.2024.104716 (PMC11413522; doi:10.1016/j.ridd.2024.104716)
Supplement: Supplementary file 2 — Supplementary material [file mmc2.docx]

| **TABLE S2. Correlation between Maternal and Child Characteristics, and Quality of Family Life (PedsQL-FIM) sum scores** | | | | | | |
| --- | --- | --- | --- | --- | --- | --- |
|  | **Total group (n=237)** | | **Developmental Disabilities (n=139)** | | **Physical Health Conditions (n=102)** | |
|  | *r* | *p* | *r* | *p* | *r* | *p* |
| **Characteristics** |  |  |  |  |  |  |
| Caregiver: age at intake | -0.17 | 0.008 | -0.20 | 0.021 | -0.08 | 0.405 |
| Caregiver: level of education | 0.26 | 0.001 | 0.25 | 0.003 | 0.33 | 0.001 |
| Child: age at intake | -0.15 | 0.024 | -0.21 | 0.013 | 0.10 | 0.310 |
| Note. Depending on the scale level and/or distribution of the variable, a Pearson correlation coefficient or Kendall's tau coefficient is used. *r =* correlation coefficient, *p* = p-value. | | | | | | |
